# Supplementary figures and images for: Calcium signaling regulates apoptosis-induced proliferation in Drosophila
Source: PLoS Biol. 2026 Jan 20;24(1):e3003607. doi: 10.1371/journal.pbio.3003607 (PMC12829959; doi:10.1371/journal.pbio.3003607)

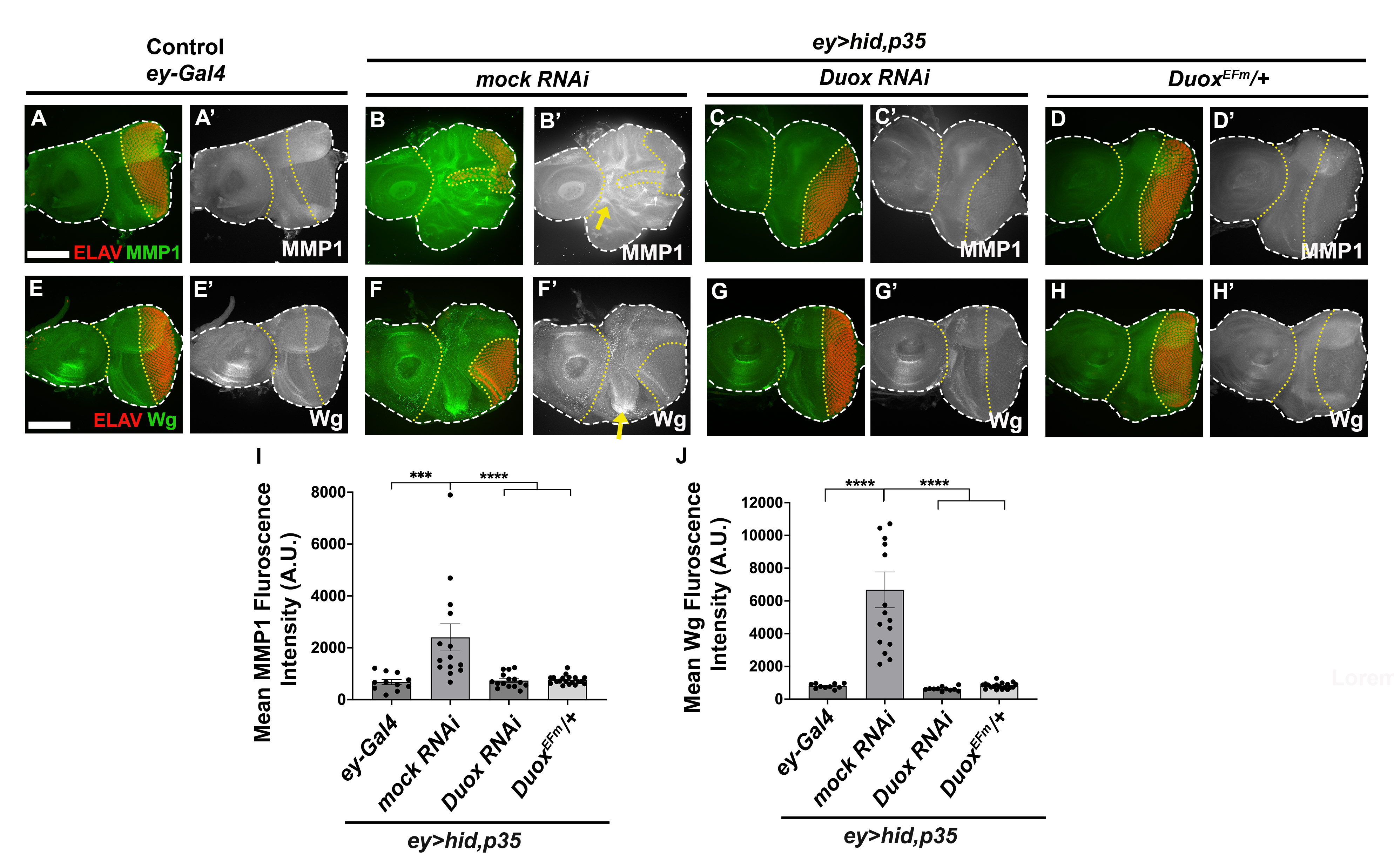

Supplement: S1 Fig — (Related to Fig 1). Disc boundaries are outlined with white dashed lines, and yellow dotted lines delineate ey-Gal4-expressing areas of the eye discs. Scale bars represent 50 μm (A–H). (A–D) Confocal images of third instar larval eye imaginal discs of control ey-Gal4 (A, A′), undead (ey>hid,p35) discs expressing mock (Luciferase) RNAi (B, B′), UAS-Duox RNAi (C, C′) and DuoxEFm mutant (D, D′) immunolabeled with MMP1 (a JNK activity marker) and ELAV antibodies. The suppression of the overgrowth phenotype by UAS-Duox RNAi and DuoxEFm (Fig 1) correlates with reduced JNK activity (MMP1; green in A–D; gray in A′, D′; see yellow arrow) and normalization of eye disc patterning as visualized by ELAV labeling (red). (E–H) Confocal images of third instar larval eye imaginal discs of control ey-Gal4 (E, E′), undead (ey>hid,p35) discs expressing mock (Luciferase) RNAi (F, F′), UAS-Duox RNAi (G, G′), and DuoxEFm mutant (H, H′) immunolabeled with Wingless (Wg) and ELAV antibodies. The suppression of the overgrowth phenotype by UAS-Duox RNAi and DuoxEFm (Fig 1) correlates with reduced Wg expression (green in E–H; gray in E′–H′; see yellow arrow) and eye disc patterning was normalized as seen by ELAV labeling (red). (I) Quantification of the MMP1 fluorescence levels in (A–D). Data from n = 11 (ey-Gal4), 14 (mock RNAi), 15 (UAS-Duox RNAi), and 19 (DuoxEFm) discs were analyzed in three independent experiments. A.U.—arbitrary units. (J) Quantification of the Wg fluorescence levels in (E–H). Data from n = 10 (ey-Gal4), 16 (mock RNAi), 11 (UAS-Duox RNAi), and 19 (DuoxEFm) discs were analyzed in three independent experiments. A.U.—arbitrary units. The data underlying the graphs shown in this figure can be found in S1 Data. (TIF) [file pbio.3003607.s001.tif]

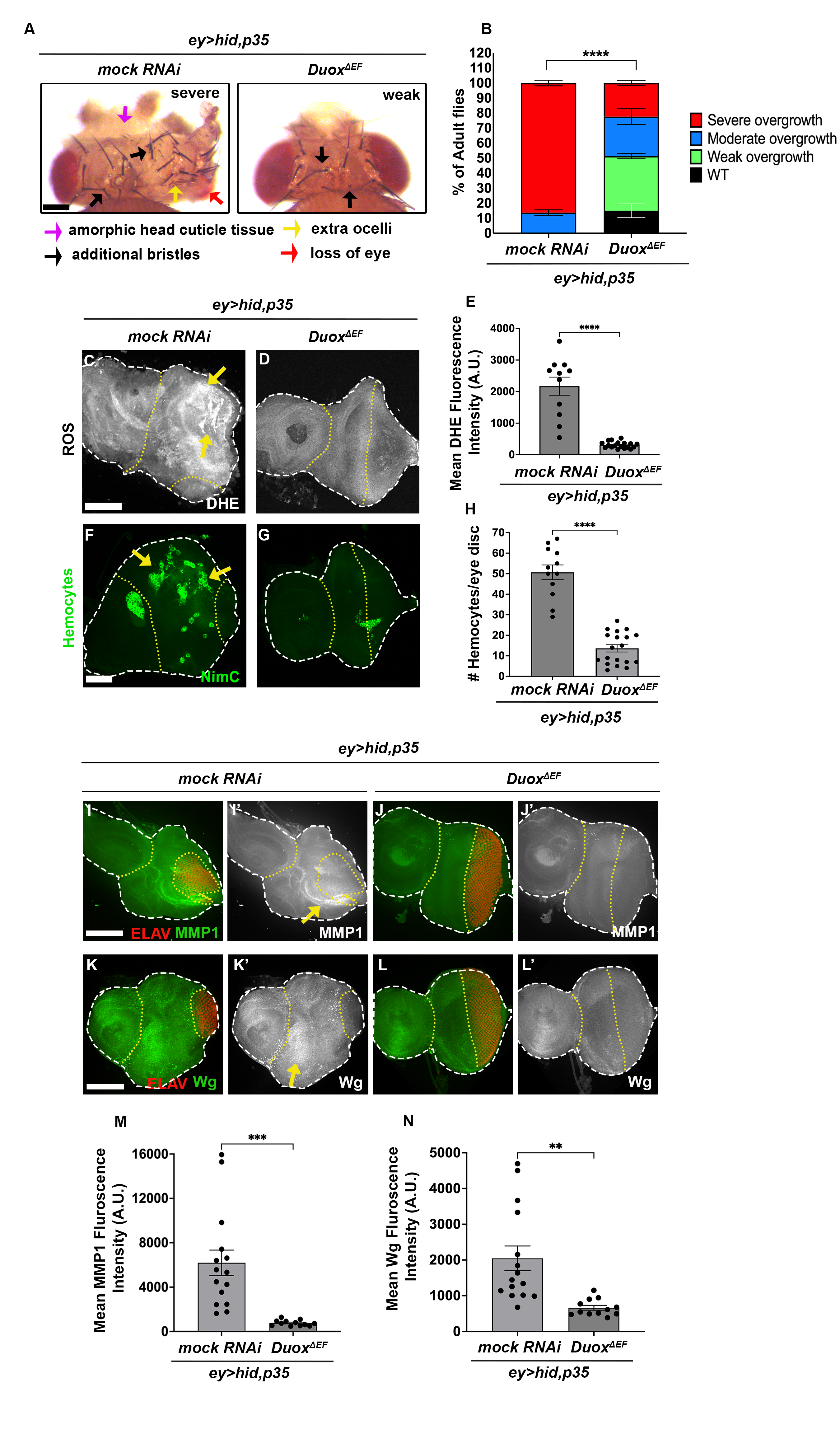

Supplement: S2 Fig — (Related to Fig 1). Disc boundaries are outlined with white dashed lines, and yellow dotted lines delineate ey-Gal4-expressing areas of the eye discs. Scale bars: 100 μm (B) and 50 μm (C, D, F, G, I, J, K, and L). (A) Representative examples of a severely overgrown head of ey>hid,p35 flies expressing mock (Luciferase) RNAi (left) and the suppressed overgrowth of ey<hid,p35 flies expressing UAS-Duox RNAi (right). Arrows point to amorphic tissues (purple arrow), additional bristles (black arrows), and ocelli (yellow arrow), and reduced eye tissue (red arrow). (B) Quantification of the suppression of head overgrowth of adult ey>hid,p35 flies by expression of UAS-DuoxΔEF. Progeny was scored as wild type (WT) (black bars), weak (green bars), moderate (blue bars), or severely overgrown (red bars) according to the classification in Fig 1A. n = 100 flies counted per genotype in three independent experiments. (C, D) Confocal images of third instar larval ey>hid,p35 discs expressing mock (Luciferase) RNAi (C) and UAS-DuoxΔEF (D) labeled for ROS with dihydroethidium (DHE) dye. The yellow arrows indicate DHE-positive cells. (E) Quantification of the DHE fluorescence levels in (C, D). Data from n = 11 (mock RNAi) and 18 (UAS-DuoxΔEF) discs were analyzed in three independent experiments. A.U.—arbitrary units. (F, G) Confocal images showing hemocytes labeled with the plasmatocyte-specific anti-NimC antibody in third instar larval ey>hid,p35 discs expressing mock (Luciferase) RNAi (F) and UAS-DuoxΔEF (G). Yellow arrows indicate hemocytes. (H) Quantification of the number of hemocytes in (F, G). Data from n = 12 (mock RNAi) and 19 (UAS-DuoxΔEF) discs were analyzed in three independent experiments. (I, J) Confocal images of third instar larval eye imaginal discs of undead (ey>hid,p35) discs expressing mock (Luciferase) RNAi (I, I′), and UAS-DuoxΔEF (J, J′) immunolabeled with MMP1 (a JNK activity marker) and ELAV antibodies. The suppression of the overgrowth phenotype by UAS-DuoxΔEF ( [file pbio.3003607.s002.tif]

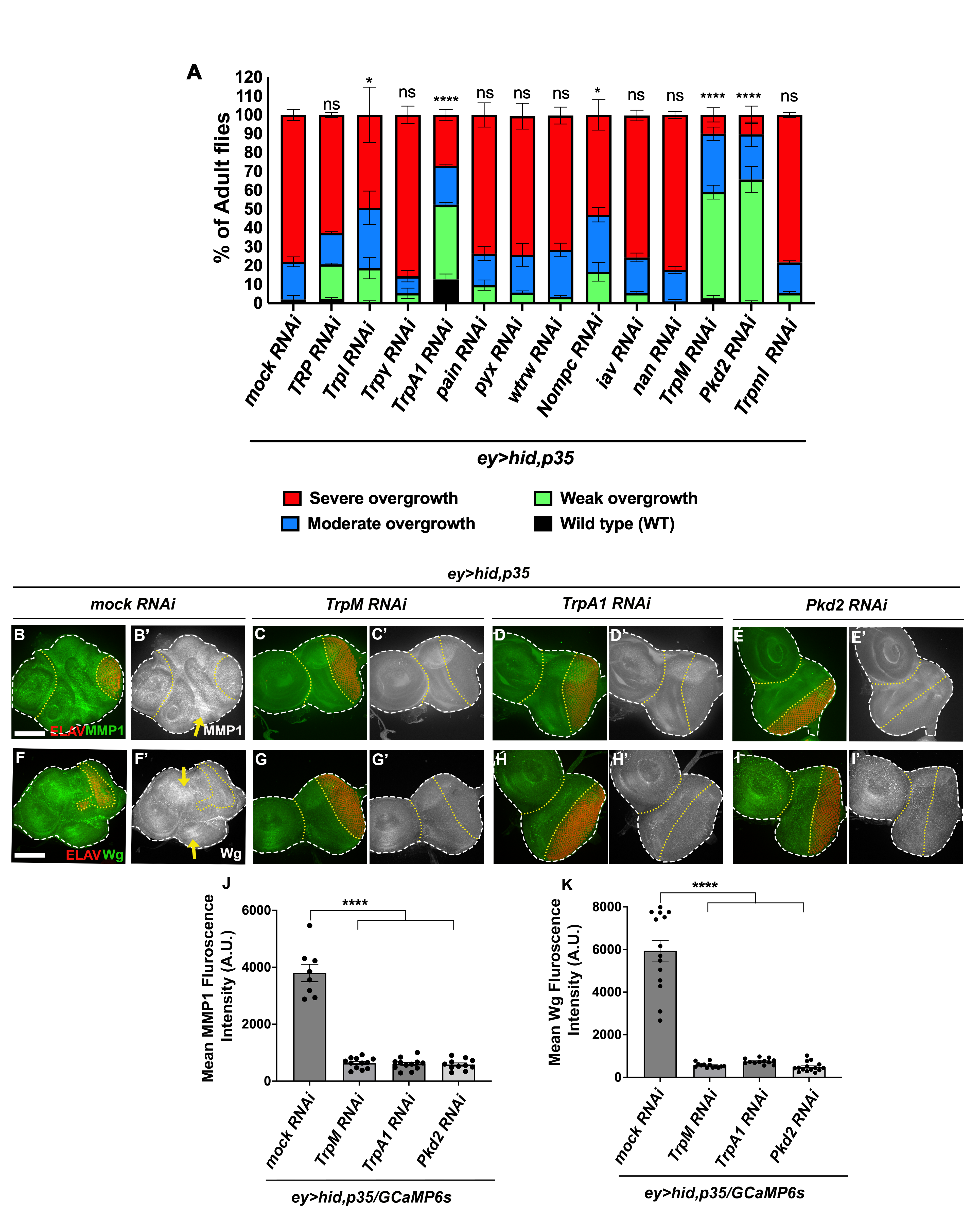

Supplement: S4 Fig — (Related to Fig 3). (A) Summary of the suppression screen targeting all 13 TRP channels in the D. melanogaster genome. Quantification of overgrowth suppression of adult ey>hid,p35 fly heads includes RNAi knockdown of control mock (Luciferase), TRP, TRPL, TRPγ, TrpA1, Painless (Pain), Pyrexia (pyx), Water witch (wtwr), NompC, Inactive (iav), Nanchung (nan), TrpM, Pkd2, and TRPML genes. Progeny was classified as wild type (wt) (black bars), weak (green bars), moderate (blue bars), or severe overgrown (red bars) based on criteria in Fig 1A. n = 100 flies counted per genotype in three independent experiments. TrpM, TrpA1, and Pkd2 showed the strongest suppression and were selected for further characterization. Disc boundaries are outlined with white dashed lines, and yellow dotted lines delineate ey-Gal4-expressing areas of the eye discs (B–E and F–I). In all panels, Scale bars are 50 μm. (B–E) Confocal images of undead third instar larval (ey>hid,p35) eye discs expressing mock (Luciferase) RNAi (B, B′), UAS-TrpM RNAi (C, C′), UAS-TrpA1 RNAi (D, D′), and UAS-Pkd2 RNAi (E, E′) immunolabeled with MMP1 (JNK marker) and ELAV antibodies. The strong MMP1 labeling in ey>hid,p35 discs (A, A′; yellow arrow) is strongly suppressed by inactivation of either TRP channel (B–D; B′–D′). ELAV labeling (red) indicates normalization of eye disc patterning upon TRP channel knockdown (B–E, red). (F–I) Confocal images of undead third instar larval (ey>hid,p35) eye discs expressing mock (Luciferase) RNAi (F, F′), UAS-TrpM RNAi (G, G′), UAS-TrpA1 RNAi (H, H′), and UAS-Pkd2 RNAi (I, I′) immunolabeled with Wg (green in F–I; gray in F′–I′; see yellow arrow) and ELAV antibodies (red in F–I). (J) Quantification of the MMP1 fluorescence levels in (A–D). Data from n = 8 (mock RNAi), 12 (UAS-TrpM RNAi), 12 (UAS-TrpA1 RNAi), and 11 (UAS-Pkd2 RNAi) discs were analyzed in three independent experiments. A.U.—arbitrary units. (K) Quantification of the Wg fluorescence levels in (E–H). Data from n = 14 (moc [file pbio.3003607.s004.tif]

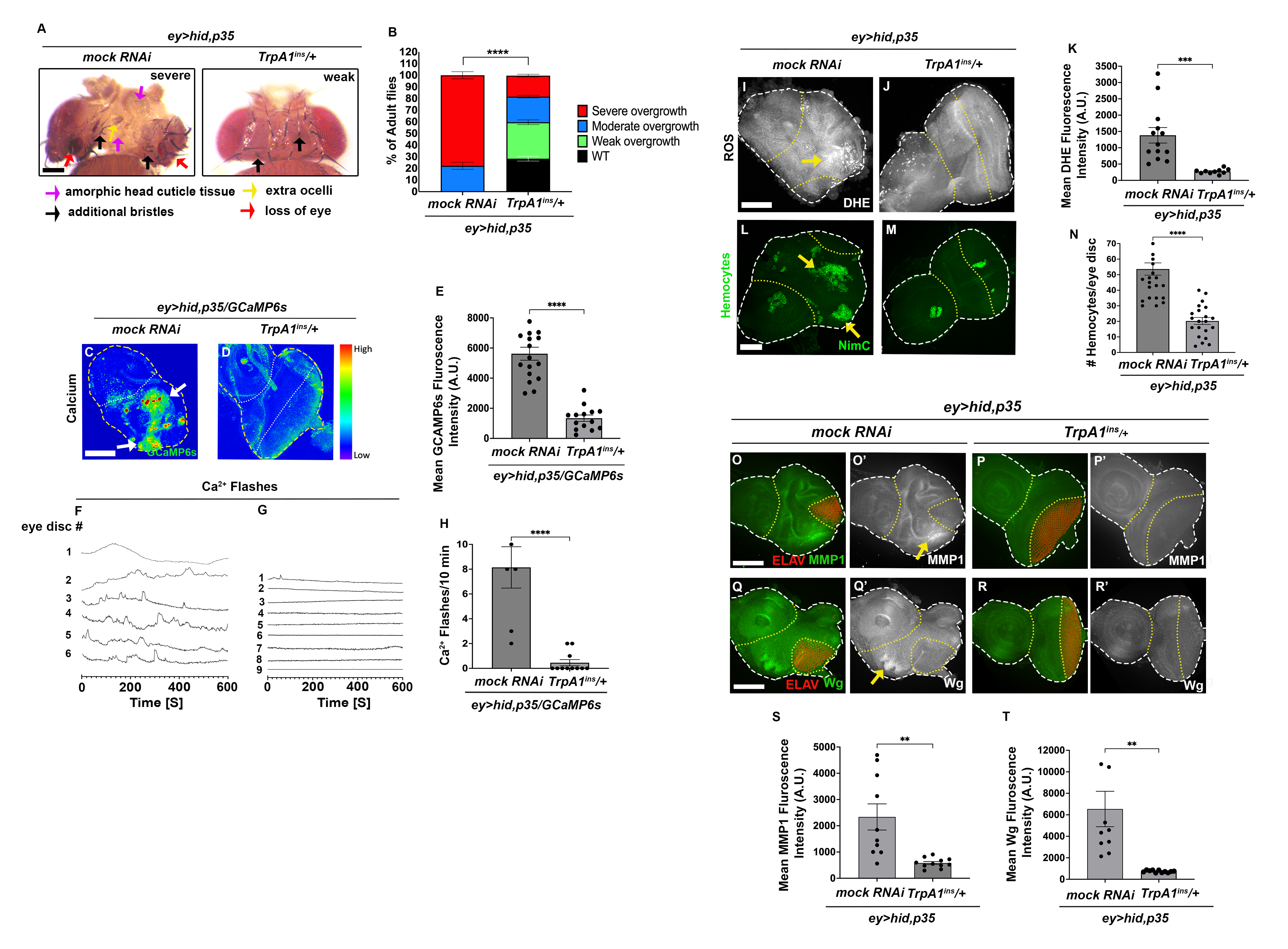

Supplement: S5 Fig — (Related to Fig 3). Disc boundaries are outlined with white dashed lines, and yellow dotted lines delineate ey-Gal4-expressing areas of the eye discs (B–E and F–I). Scale bars: 100 μm (A) and 50 μm (C, D, I, J, L, M, O, P, Q, and R). (A) Representative examples of a severely overgrown head of ey>hid,p35 flies expressing mock (Luciferase) RNAi and the suppressed overgrowth by TrpA1ins/+. Arrows point to amorphic tissues (purple arrow), additional bristles (black arrows) as well as ocelli (yellow arrow) and loss of eye tissue (red arrows) in ey>hid,p35 expressing mock (Luciferase) RNAi, while black arrows in TrpA1ins/+ point to one or two extra bristles. (B) Quantification of the dominant suppression of head overgrowth of adult ey>hid,p35 flies by heterozygous TrpA1ins/+. Progeny was scored as wild type (wt) (black bars), weak (green bars), moderate (blue bars), or severe overgrowth (red bars) according to the classification in Fig 1A. n = 100 flies counted per genotype in three independent experiments. (C, D) Confocal images of third instar larval ey>hid,p35 eye imaginal discs expressing the Ca2+ reporter GCaMP6s with and without TrpA1ins/+. White arrows point to high Ca2+ levels. (E) Quantification of the cytosolic Ca2+ levels in (C, D) via measuring GCaMP6s fluorescence intensity. Data from n = 17 (mock RNAi) and 14 (TrpA1ins/+) discs were analyzed in three independent experiments. A.U.—arbitrary units. (F, G) Representative Ca2+ traces of eye imaginal discs expressing GCaMP6s obtained by time-lapse confocal imaging (600 frames, 1-second intervals). Each line represents an independent disc (numbered). For complete individual recordings of the Ca2+ traces, see S6 Fig. The numbers of Ca2+ flashes are strongly reduced by TrpA1ins/+ (S4 and S10 Movies). Quantification shown in (H). Genotypes: (F) ey>hid,p35/GCaMP6s/Luciferase RNAi (n = 6); (G) ey>hid,p35/GCaMP6s/TrpA1ins/+ (n = 9). (H) Quantification of Ca2+ flashes in (E, F). Data from n = 7 (mock RNAi) and 11 (TrpA1i [file pbio.3003607.s005.tif]

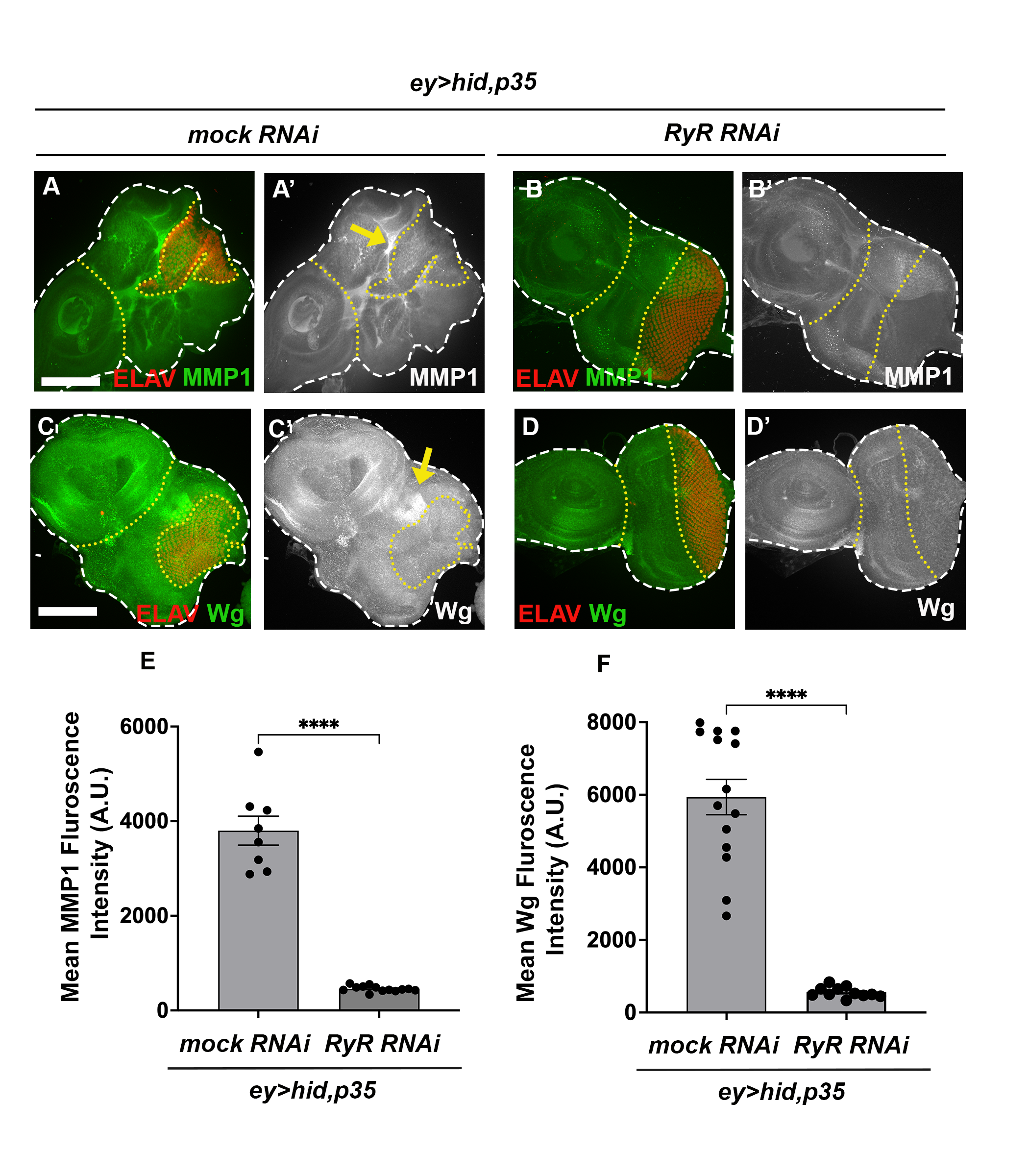

Supplement: S7 Fig — (Related to Fig 4). Disc boundaries are outlined with white dashed lines, and yellow dotted lines delineate ey-Gal4-expressing areas of the eye discs (A, B, A′, B′, and C, D, C′, D′). In all panels, Scale bars represent 50μm. (A, B) Confocal images of third instar larval eye imaginal discs of undead (ey>hid,p35) discs expressing mock (Luciferase) RNAi (A, A′) and UAS-RyR RNAi (B, B′) immunolabeled with MMP1 and ELAV antibodies. MMP1 labeling (green in A, B; gray in A′, B′; see yellow arrow) is strongly reduced by UAS-RyR RNAi. ELAV labeling (red) indicates normalization of eye disc patterning by UAS-RyR RNAi. (C, D) Confocal images showing third instar larval eye imaginal discs of undead (ey>hid,p35) discs expressing mock (Luciferase) RNAi (C, C′) and UAS-RyR RNAi (D, D’′ labeled with Wingless (Wg) and ELAV antibodies. Wg labeling (green in C, D; gray in C′, C′; see yellow arrow) is strongly reduced by UAS-RyR RNAi. ELAV labeling (red) indicates normalization of eye disc patterning. (E) Quantification of the MMP1 fluorescence levels in (A, B). Data from n = 8 (mock RNAi) and 13 (UAS-RyR RNAi) discs were analyzed in three independent experiments. A.U.—arbitrary units. (F) Quantification of the Wg fluorescence levels in (C, D). Data from n = 14 (mock RNAi), and 11 (UAS-RyR RNAi) discs were analyzed in three independent experiments. A.U.—arbitrary units. The data underlying the graphs shown in this figure can be found in S1 Data. (TIF) [file pbio.3003607.s007.tif]

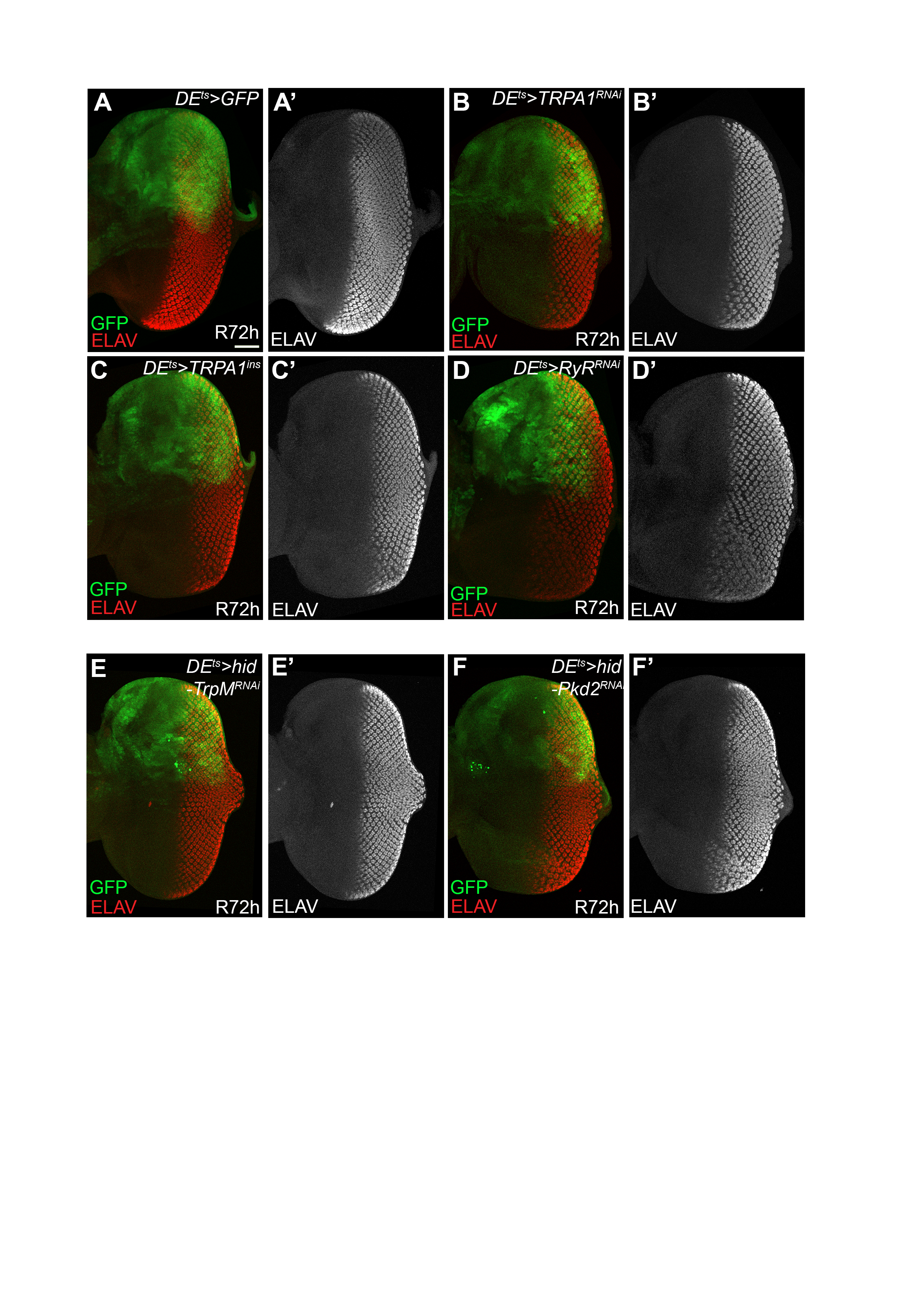

Supplement: S8 Fig — (Related to Fig 5). (A) Control DEts>GFP disc at R72h. Following the 12 h temperature shift that induces GFP expression (green) in the dorsal half of the eye imaginal discs (see experimental protocol in Fig 5A), photoreceptor patterning appears normal as shown by ELAV labeling (red in A, gray in A′). Scale bar: 50 μm. (B) DEts>GFP,TrpA1 RNAi disc at R72h. TrpA1 RNAi does not affect photoreceptor development following 12 h induction at 30 °C and 72 h recovery at 18 °C (Fig 5A). ELAV staining appears normal (red in B, gray in B′). GFP expression (green) indicates that transgenes have been induced. (C) DEts>GFP; TrpA1ins disc at R72h. TrpA1ins/+ does not affect photoreceptor development following 12 h incubation at 30 °C and 72 h recovery at 18 °C (Fig 5A). ELAV staining appears normal (red in B, gray in B′). (D) DEts>GFP,RyR RNAi disc at R72h. RyR RNAi does not affect photoreceptor development following 12 h induction at 30 °C and 72 h recovery at 18 °C (Fig 5A). ELAV staining appears normal (red in D, gray in D′). GFP expression (green) indicates that transgenes have been induced. (E, F) DEts>hid eye discs at R72h expressing UAS-TrpM RNAi (E) and UAS-Pkd2 RNAi (F) show complete recovery, with all examined discs (n = 20 for E; n = 10 for F) displaying restored ELAV expression (red in E, F; gray in E′,F′). GFP expression (green) confirms induction of transgenes. (TIF) [file pbio.3003607.s008.tif]
