# Supplementary material for: Calcium signaling regulates apoptosis-induced proliferation in Drosophila
Source: PLoS Biol. 2026 Jan 20;24(1):e3003607. doi: 10.1371/journal.pbio.3003607 (PMC12829959; doi:10.1371/journal.pbio.3003607)
Supplement: Attachment — Submitted filename: Response to the reviewers comments.docx [file pbio.3003607.s022.docx]

**POINT-by-POINT response to reviewer’s comments**

We would like to sincerely thank the reviewers for the time and effort they devoted to evaluate our manuscript and for their constructive comments that helped us improve the work. We are pleased to report that we have carefully addressed all concerns and suggestions raised. In the course of revision, we have added substantial new data that significantly strengthen the manuscript. Most notably, we generated and analyzed a new mutant in the endogenous *Duox* gene**,** termed ***Duox^EFm^***, in which two invariant glutamate residues in the EF-hand motifs are replaced by glutamines, thereby abolishing Ca²⁺ binding. This new reagent directly confirms that DUOX activation is Ca²⁺ dependent and provides strong mechanistic support for our central conclusion. In addition, we performed new experiments using the ***DE^ts^*>*hid* genuine ablation system**, which independently validates the important roles of TrpA1 and the RyR in genuine AiP and regenerative responses to tissue loss. Finally, we added a **new transcriptional analysis**, showing that several Ca²⁺ channels are transcriptionally upregulated in undead cells, further supporting their physiological relevance in AiP. Together, these additions not only address the comments of the reviewers but also provide a much more comprehensive and compelling case for the role of Ca²⁺ signaling in DUOX activation and AiP.

In the following, we provide a detailed point-by-point response to the reviewers’ comments (our responses in blue).

**Rev. 1:**
The report by Suthar and Bergmann addresses the molecular mechanisms regulating the process of apoptosis-induced proliferation (AiP). Building on previous work from the Bergmann laboratory, where several seminal discoveries of the basic biology regulating AiP, the authors now explore the mechanisms leading to the activation of Duox, one of the critical regulators of AiP. The authors show that, in agreement with its protein and domain structure, Duox is activated by Ca2+ and this activation is dependent on Dronc function. The authors also show that the Ca2+ is mobilised from different sources. Ca2+ is internalised from extracellular sources via specific Ca2+ channels and mobilised from the ER to activate Duox. The authors suggest that Ca2+ is therefore essential for AiP.

In general, experiments and data are well presented and appropriately controlled. The results obtained by the authors provide an extra layer of complexity regarding the process of AiP and will be of interest to groups studying apoptosis and regeneration, among others. Comments on the manuscript are shown below.

Major points:
Figure 2: Regarding the analysis of Ca2+ signalling, is there a pattern in the distribution of the Ca2+ flashes? Do they primarily occur in the ey-Gal4-expressing areas or are the surrounding areas also affected?

**Response**: The Ca²⁺ flashes are restricted to the *ey-Gal4*–expressing areas and we do not observe them in the surrounding tissue. Within the *ey-Gal4* domain, the flashes occur without an obvious spatial pattern. We have clarified this point in the revised manuscript.

Related to the point above and given that there is an amplification loop of AiP signalling, is there a role for Duox in the regulation of Ca2+ flashes or AiP-mediated Ca2+ signalling?

**Response**: Yes, indeed, our data directly support such a role. We find that DUOX is required not only downstream of Ca2+ signaling, but also for the maintenance of Ca2+ flashes through the amplification loop. Specifically, when we knock down *Duox* by RNAi, Ca²⁺ flashes in undead discs are strongly reduced or abolished, despite the presence of upstream TRP channel activity. This demonstrates that DUOX activity feeds back into Ca2+ dynamics: DUOX-generated ROS potentially activate the redox-sensitive TrpA1 channels, which sustain and amplify Ca2+ entry, thereby reinforcing Ca2+ flashes (see also step 4 in the model of Figure 6).

Regarding the role of Ca2+ channels, is there an additive or synergistic effect of modulating extracellular Ca2+ channels and RyR regarding AiP phenotypes? Is there a way to test the epistatic relationship between the different Ca2+ channel types (i.e. those controlling extracellular Ca2+ and RyR)?

**Response**: We propose that the extracellular TRP channels and the RyR have a synergistic rather than an additive relationship. This synergy is comes through the amplification of Ca²⁺ signaling via calcium-induced calcium release (CICR), which is mediated by RyR in response to the initial Ca²⁺ influx through the extracellular TRP channels. The synergistic relationship is supported by our knockdown experiments, which show that silencing any single TRP channel (TrpA1, TrpM, or Pkd2) or RyR substantially reduces cytosolic Ca²⁺ levels, Ca²⁺ flashes, and ROS production. These findings indicate that both extracellular Ca²⁺ influx and RyR-mediated CICR are essential components, which suggests a synergistic rather than merely additive interaction.

We agree with the reviewer that it would be very informative to know the epistatic relationship between these Ca²⁺ channels. However, establishing this epistatic relationship requires opposing phenotypes that can be tested against each other. Our current challenge is that loss-of-function for all these components results in the same phenotype (reduced Ca²⁺ signaling), and we lack gain-of-function phenotypes with the available reagents.

All the experiments have been performed using the "undead" cell system, which has been exploited at length in the field of AiP. However, by its nature, this is a system where AiP is prolonged and extended beyond what would normally occur in a physiological setting. Given that the authors have a TrpA1 mutant fly stock, would it be possible to conduct experiments where apoptosis is induced in a pulse (i.e. using a FLPout system) rather than sustained using "undead" cells and to compare the extent of AiP in the control situation vs the TrpA1 mutant (where AiP will be affected)? Whilst not strictly necessary, having an experiment conducted in a system beyond the "undead" model would enhance the conclusions of the study.

**Response**: We agree with the reviewer that testing beyond the undead system is important. To address this, we employed the “**genuine**” ***DE^ts^>hid*** AiP model, which is independent of p35 and that we developed previously. In this system, cells are allowed to undergo apoptosis and die, rather than being maintained in an undead state. A transient pulse of apoptosis causes tissue loss but is followed by full regeneration in controls. In contrast, when apoptosis is induced in the background of *TrpA1* RNAi, the *TrpA1* mutant, or *RyR* RNAi, the regenerative response is incomplete. These findings confirm that TrpA1 and RyR are required for regeneration in a physiological AiP setting, thereby strengthening our conclusions. These new data are shown in Fig. 5 and Supplementary Fig. S8.

Related to the point above, authors should comment on the fact that TrpA1 mutants seem to suppress the AiP phenotypes in heterozygosity. Is 50% of gene dose not sufficient to maintain TrpA1 function?

**Response**: The *ey>hid,p35* condition provides a sensitized genetic background that allows us to detect phenotypic effects from heterozygous mutations. TrpA1 is not unique in its ability to dominantly suppress *ey>hid,p35*-induced overgrowth when present in a heterozygous state. Another notable example is the JNK gene (*bsk*), which also demonstrates strong suppression when heterozygous in this background. Likewise, the *Duox^EFm^* allele described in this study acts similarly (see Figure 1). Importantly, these alleles are completely recessive when examined in isolation, and show no phenotypic effects outside of the sensitized background. Thus, the *ey>hid,p35* system reveals genetic interactions that would otherwise remain undetectable under standard conditions. This feature makes it a powerful tool for genetic screening to identify novel genes involved in AiP.

Minor points:
Figure 1A, Figure 3A, Figure S3A, Figure 4A: If possible, statistical analysis of the comparison between the genotypes should be added.

**Response**: We thank the reviewer for this suggestion. We have now performed statistical analyses and added error bars to the quantification graphs of the different overgrowth classes. Statistical significance was assessed using one-way ANOVA followed by Dunnett’s multiple comparison test. In addition, we calculated P-values based on the frequency of severely overgrown flies per genotype. These results are now included in the revised figures.

Related to Figure 1, it would be helpful to provide more information regarding the criteria to classify the different phenotypes as weak, moderate or severe overgrowth. This could potentially be included in the relevant Materials and Methods section.

**Response**: We have now added more detailed explanations of the classification criteria both in the legend to Figure 1A and in the Materials and Methods section.

Figure 1C-H, Figure 1I-N and Figure S1: Have the quantifications of the intensity of the different stainings been normalised to tissue size? Moreover, given that AiP affects neighbouring tissue, have the authors analysed and quantified the stainings in the different areas (ey-Gal4-expressing and surrounding areas) separately?

**Response**: In the case of Figure 1C-H (now Figure 1H-M) and Figure S1, we determined the mean fluorescence intensity across the entire *ey-Gal4*-expressing area which provides normalization for tissue size. In the case of Figure 1I-N (now Figure 1N-S), we quantified hemocytes across the entire eye disc to capture the full inflammatory response to the AiP condition. Regarding the second question, we have quantified the stainings only in the *ey-Gal4* expressing areas.

Are both EF hand motifs required? Have the authors created Duox transgenes where only one EF hand has been mutated? If so, do these have intermediate phenotypes?

**Response**: We thank the reviewer for this insightful question. While we have not generated transgenes with individual EF-hand deletions, previous work has shown that EF hands can act cooperatively, such that disruption of a single motif can already compromise activity, indicating functional dependence between the two motifs (reference [77] in the manuscript).

Rather than generating new transgenes with individual EF hand deletions, we used CRISPR/Cas9 technology to introduce two point mutations (one in each EF-hand motif) in the endogenous *Duox* gene, termed *Duox^EFm^* (for EF mutant). Specifically, we changed two invariant glutamate (Glu) residues to glutamine (Gln), which is known to disrupt Ca2+ binding [77]. This endogenous mutant behaves as a strong dominant suppressor of AiP-induced overgrowth, demonstrating that Ca2+ binding through the EF-hand motifs is critical for DUOX function in this context. While this approach does not directly dissect the individual contributions of each EF hand motif, it further strengthens the critical role of Ca2+ binding to the EF-hands for DUOX activity. These results are presented in Figure 1.

Related to the action of the Ca2+ channels, is it possible for the authors to specifically inhibit the different channels using chemical inhibitors to further confirm their role and potentially explain the non-redundancy issue? Alternatively, is it possible for authors to over-express (or deplete) Ca2+ regulators with the opposite effect of RyR, TRPA1, TRPM and PKD2 to further validate their results?

**Response**: We thank the reviewer for these excellent suggestions regarding further validation of the calcium channel requirements. However, regarding chemical inhibitors, while this is an interesting approach in principle, there are several practical limitations. First, many calcium channel inhibitors lack the specificity required to distinguish between closely related channel family members, which could lead to off-target effects that complicate interpretation. Second, pharmacological inhibition in the *Drosophila* system, particularly for tissue-specific effects during development, presents technical challenges in achieving appropriate dosing and temporal control compared to genetic manipulation. Third, the availability of well-characterized, specific inhibitors for these channels in *Drosophila* is limited.

With respect to genetic approaches, our study focused on loss-of-function analyses to establish the requirement of *TrpA1*, *TrpM*, *Pkd2*, and *RyR*. Complementary strategies such as overexpression of Ca²⁺ regulators with opposing activities could in principle provide additional insights, but they may also introduce complexity in interpretation, as Ca2+ homeostasis is tightly regulated and non-physiological perturbations often trigger pleiotropic effects. For these reasons, we concentrated on the well-established knockdown and mutant analyses, which provide clear evidence for the essential and non-redundant roles of these channels in AiP

Line 215: Typo. S1I should be replaced with S2I.

**Response**: Thank you for picking up this typo. It has been corrected.

Line 216: Typo S1J should be replaced with S2J.

**Response**: Thank you for picking up this typo. It has been corrected.

Figure 4G: x-axis labels are covered in figure panel.

**Response**: This panel (now panel 4H) has been corrected.

Line 554: Reference 39 is listed as in press but the correct citation should be provided.

**Response**: The citation of the reference has been updated.


Rev. 2:
The manuscript titled "Role of Calcium Signaling in Apoptosis-induced Proliferation in Drosophila" by Suthar and Bergmann explores the role of cytosolic calcium influx as a crucial event for DUOX activation during apoptosis-induced proliferation (AiP), using the Drosophila eye disc as a model system. This study builds upon previous work demonstrating that activation of the caspase Dronc leads to DUOX activation in "undead" cells, which produces reactive oxygen species (ROS), which recruits immune cells to activate JNK signaling and downstream mitogenic factors that promote proliferation of surrounding cells. In this work, the authors identify the calcium channels - TRPA1, TRPM, PKD2, and RyR - that allow Ca2+ entry into the cytosol when apoptosis is inhibited by p35, activating DUOX via its EF-hand calcium-binding motifs to drive AiP.

To establish the role of these channels, the authors employ a comprehensive suite of genetic tools, including RNA interference, CRISPR-Cas9-mediated knockouts, and mutants. These complementary approaches offer compelling evidence linking calcium channel activity to DUOX activation. However, the authors point out that calcium regulation of DUOX has been previously described, and identification of these channels does not explain how the caspase Dronc activates this pathway. While this manuscript is essentially a genetic screen paper, the screen itself is not presented. Furthermore, the duplication of controls throughout the manuscript raises concerns about the data itself. Thus, substantial work remains before this manuscript is suitable for a journal with a broad readership such as Plos Biology.

Major concerns:
1. Line 192-194: The authors mention a screen was conducted of 13 TRP channel genes. This paper is a genetic screen paper in which the screen itself is not presented. The authors should present the complete screen. How do we know that perturbation of any calcium channel will not have the same effect, and that the ones presented are unique in their role in AiP?

**Response**: We have now included the complete results of the TRP channel screen in Supplementary Figure S4A. These data demonstrate that, among all 13 TRP channels tested, TrpA1, TrpM, and Pkd2 show strong suppression of AiP phenotypes, confirming that their contribution is specific and not a general property of TRP channel perturbation.

2. The new finding in this paper is identification of the specific calcium channels that act downstream of Dronc to promote Duox activity, as the fact that calcium activates Duox is known. This finding is assuming that presentation of the full screen as requested in point 1 will confirm that the effect on DUOX activity is specific to these four channels. Identification of how Dronc activates these channels would substantially increase the broad applicability of these findings and interest in this work.

**Response**: We agree with the reviewer's insightful point regarding the mechanism of Dronc-mediated channel activation. This question is of considerable interest to us as well. Direct activation seems unlikely, as caspase-mediated cleavage typically results in protein inactivation rather than activation. It therefore remains unclear whether Dronc interacts with these channels through proteolytic activity or through non-proteolytic mechanisms such as scaffolding or binding functions, or an indirect signaling mechanism. We have included the potential mechanisms by which Dronc may activate the TRP channels in the Discussion section.

3. The controls presented in the figures are identical for each figure - the same disc images are used throughout, sometimes tilted and sometimes not, and the graphs make it clear that the same measurements are graphed for the controls for different experiments. Thus, it appears the controls were done only once, and the numbers re-used for subsequent experiments without running the control side-by-side with the mutant or knockdown being tested. This would render the result invalid, as controls must be performed alongside the experimental condition for immunofluorescence or DHE staining.
a. DHE: 1D, 3N, 4H, S3H, are the same disc. 1H, 3D, 4J, S3J use the same data for the mock RNAi genotype.
b. NimC: 1J, 3R, 4K, S3K are the same disc, slightly rotated. 1N, 3E, S3M use the same data for the mock RNAi genotype.
c. GCamp: 2I, 3F, 4B, S3B are the same disc. 2K and 3B may be the same data plotted slightly differently. 2K, 4D, and S4D are the same data for the mock RNAi genotype.
d. Mmp1: S1B, S2A, S3N, and S4A are the same disc. S1K, S2I, S3R, and S4E are the same data for the mock RNAi genotype.
e. Wg: S1F, S2E, S3P, and S4C are the same disc. S1J, S2J, S3S, and S4S have the same data for the mock RNAi genotype.
Therefore, these experiments should all be repeated with side-by-side controls to confirm the results.

**Response**: We thank the reviewer for noting this issue. We would like to clarify that all of our experiments were always performed with side-by-side controls. However, in preparing the figures, the reiterative use of the same control panel across several figures reflected the fact that the results of the control experiments were consistently reproducible across independent experiments. We recognize that this may have given the unintended impression that controls were not run in parallel, and we have now replaced the control panels with images from the actual side-by-side experiments corresponding to each figure cited. The only instances where the same control panels appear are in Figures 3K,O and 4I,L, as well as S4B,F and S7A,F, since the TRP and RyR experiments were performed simultaneously along with the control, hence we used the same control discs in these figures.

4. In Figure 1B, the four panels depict the extent of the overgrown phenotype, which includes a range of phenotypes relating to bristles, ocelli, eye size etc. The arrows should be changed to different markers pointing at different phenotypes. Importantly, each specific phenotype should be separately quantified to make its prevalence more apparent.

**Response**: We appreciate this helpful suggestion. In the revised figure (now Figure 1A), we have improved the visual presentation by using distinct markers (different colored arrows) to highlight the various phenotypic features such as bristles, ocelli, and eye size. In addition, we now describe in more detail the criteria used to define each overgrowth category in both the figure legend and the Materials and Methods section. With respect to the reviewer’s final point, we would like to note that the individual phenotypic categories (weak, moderate, severe) are already quantified separately in Figure 1B.

5. Line 199-206: The authors show an interesting finding, that inactivating any single
channel results in loss of GCaMP6s activity, suggesting these channels are non-redundant. However, no experiments were designed to probe the reason behind the fact that all appear necessary and none appear sufficient to activate Duox. The explanation provided in the discussion is unconvincing. If these channels have their own unique inputs, why would inactivating any one channel abolish all or most cytosolic calcium?

**Response**: We agree with the reviewer that the non-redundant role of the TRP channels is a very surprising result and we also were equally perplexed when we saw these results. Importantly, TrpM, TrpA1, and Pkd2 belong to different TRP channel sub-families with distinct structural and functional properties, suggesting that their requirement may be non-redundant due to unique sub-family-specific contributions. Since our original explanation in the Discussion was deemed unconvincing, we have expanded the text in the Discussion to consider alternative possibilities. These include the idea that the channels act as an interdependent complex or signaling network, that they function in a sequential or feedback-dependent manner, or that each contributes a necessary portion of Ca²⁺ influx such that loss of any one prevents reaching the threshold required for DUOX activation. Another possibility is that RyR activation requires the coordinated input from all TRP channels, such that disruption of one impairs CICR and abolishes cytosolic Ca²⁺ activity. While the precise mechanism remains unresolved, we believe these considerations provide a stronger framework for interpreting this unexpected non-redundancy.

We have revised the Discussion accordingly and hope that these explanations address the concerns of the reviewer. We would also like to point out that there are several precedents in the literature describing similar non-redundant channel functions in other biological contexts, and we have incorporated these examples into the Discussion to support the general plausibility of our findings.

6. In the discussion (line 285-287), the authors mentioned some TRP channels were upregulated under certain conditions like cancer. Are they upregulated in the context of AiP as well?

**Response**: We thank the reviewer for raising this point. We addressed it by performing qRT-PCR analysis (Figure 4O). The results show that *RyR* is strongly upregulated in undead discs, while *TrpA1* and *TrpM* are modestly upregulated. In contrast, *Pkd2* expression remains unchanged. These findings indicate that at least some of the Ca²⁺ channels required for AiP are transcriptionally induced in this context.

7. In Fig 2C, there seems to be more GCaMP6s activity in the antennal region of the disc upon generation of undead cells, despite the system being restricted to ey+ cells. A similar effect is seen in the RyR RNAi disc (Fig 4C). The authors should explore why this is happening.

**Response**: We appreciate the reviewer noting these observations. Upon closer examination, we found that the apparent signals in the antennal region were minor and not consistently observed across samples. To avoid potential confusion, we have replaced the affected panels with more representative images that reflect the consistent results obtained in multiple independent discs. Importantly, these adjustments do not affect our conclusions.

8. In Figure S2, Wg and MMP1 also seem to be upregulated in the antennal region, despite the undead cells restricted to the eye disc anterior to the furrow. The authors should quantify Wg and Mmp1 in the antennal discs, and if there is truly elevated expression non-autonomously, explain why.

**Response**: Similar to the previous point, after careful examination, we conclude that the effects in question are relatively minor. Regarding Wg signaling specifically, it's important to note that the antennal disc normally exhibits developmental Wg expression that is independent of AiP. To avoid confusion, we have replaced these panels with representative images that more accurately demonstrate the consistent patterns observed across our experiments. Quantification was always performed in the eye disc region where AiP occurs (not in the antennal region), and these adjustments do not affect our results or conclusions.


Minor comments:

1. Line 53, add a period after caspases.

**Response**: Thank you for catching this oversight. The period has been added.

2. In figure 1A, a control for ey>hid,p35 without a mock RNAi should be added, to ensure activating the RNAi machinery does not itself cause any overgrowth.

**Response**: We thank the reviewer for this suggestion. We have now included the *ey>hid,p35* control with mock RNAi in Figure 1B (previously Figure 1A), which confirms that activation of the RNAi machinery alone does not cause overgrowth. We used Luciferase RNAi is as the mock RNAi.

3. Figure 1A-B: The examples of the phenotypes should be presented before the quantifications.

**Response**: We have switched the order of Figure 1A and 1B.

4. Figure 1: The figure legend stated that the scale bars for C-G are 50 microns and the scale bars for I-M are 100 microns, but the discs in I-M do not look half the size of the discs in C-G. This same concern exists in several other figures.

**Response**: This discrepancy arises from the use of two different confocal microscopes during data collection, which might result in small differences in disc size. However, the scale bars shown in each panel are accurate (now they are all 100μm), and within each group of panels (now H-L or N-R), images were acquired with the same microscope and the same settings to ensure internal consistency. All quantitative comparisons were performed within the same imaging set-up, so this does not affect the validity of the results.

5. In Figure 1H and N, some bars are incorrectly labeled as "Doux". The labels are too small to read here and in all graphs throughout the other figures.

**Response**: Thank you for catching these typos. We have corrected them and made the labels bigger, too.

6. Figure 1H: The legend states that 6 discs were obtained from three experiments. Two discs per experiment is a very low yield - can the authors explain?

**Response**: We thank the reviewer for pointing this out. We have now increased the number of quantified discs to 16. Moreover, these data were moved to Suppl Figure S2.

7. Figure 2 E-G and other similar panels - does each line represent a different disc? The Y-axis is not labeled (eg Fig2E-G, L etc.)

**Response**: Yes, each line represents an individual eye disc, as now stated in the revised figure legends. To improve clarity, we have also added two new supplementary figures (S3 and S6) that display the complete Ca²⁺ traces from the time-lapse recordings, with each disc shown as a separate panel depicting fluorescence intensity over time.

8. Figure 3 - show the examples of the phenotypes and then show the quantification. Otherwise, the figure panels are called out of order in the text.

**Response**: We have revised Figure 3 so that representative phenotype images are now shown first, followed by the corresponding quantifications.

9. Figure S3 was called before Figure S2; re-arrangements need to be done accordingly.

**Response**: We have reordered the figures to avoid confusion.


10. Figure 4, The images have no scale bars, and it does not seem accurate that K-L are a different scale from H-I.

**Response**: Scale bars were added.

11. Figure 4G the bottom of the graph is masked or incomplete.

**Response**: This issue has been corrected.

12. Figure S3: The images have no scale bars.

**Response**: Scale bars have been added.

13. Figure S3: the number 1055 is hovering above panel D

**Response**: Not sure what happened there, but the number has been removed.

14. Videos should all be in MP4 format for universal access

**Response**: All movies are now available in MP4 format.
