# Supplementary material for: Calcium signaling regulates apoptosis-induced proliferation in Drosophila
Source: PLoS Biol. 2026 Jan 20;24(1):e3003607. doi: 10.1371/journal.pbio.3003607 (PMC12829959; doi:10.1371/journal.pbio.3003607)
Supplement: Attachment — Submitted filename: Point-by-point response to reviewers comments.docx [file pbio.3003607.s023.docx]

**PBIOLOGY-D-25-00697R2**

**Reviewers' comments**
Rev. 1: Paulo Ribeiro
The report by Suthar et al. addresses the molecular mechanisms regulating the process of apoptosis-induced proliferation (AiP). Building on previous work from the Bergmann laboratory, where several seminal discoveries of the basic biology regulating AiP, the authors now explore the mechanisms leading to the activation of Duox, one of the critical regulators of AiP. The authors show that, in agreement with its protein and domain structure, Duox is activated by Ca2+ and this activation is dependent on Dronc function. The authors also show that the Ca2+ is mobilised from different sources. Ca2+ is internalised from extracellular sources via specific Ca2+ channels and mobilised from the ER to activate Duox. The authors suggest that Ca2+ is therefore essential for AiP.

The revised manuscript is greatly improved, and the authors strived to address all the major points raised by the reviewers. Importantly, the new data provided enhances and further supports the hypothesis that Ca2+ signalling plays an important role in AiP. The use of a genetic mutant of Duox that is predicted to be unable to bind Ca2+ constitutes an additional genetic system to address the role of Duox and provides additional evidence beyond the use of the dominant-negative version of Duox. The new Duox allele further supports the proposed molecular mechanism presented by the authors. Moreover, the authors also present data in a genetic system that does not result in the long-term accumulation of "undead" cells and their experiments reveal that the mechanism uncovered in this report is likely to be important in more physiological conditions, due to the fact that there was incomplete tissue regeneration when Ca2+ channel function was affected.

Experiments and data are well presented and appropriately controlled. New figure panels are more extensive and provide indication of statistical analyses proposed by the reviewers. Methods include additional information important for clarification of specific points that could be confusing to a more general audience.

In general, the manuscript is vastly improved, includes further evidence supporting the main conclusions of the report and the level of evidence provided is now supported by additional statistical analyses. The data will be of interest to research groups studying apoptosis and regeneration, among others. I support its publication in PLoS Biology.

**Response**: Thank you very much for your kind words.


Rev. 2:
The manuscript titled "Role of Calcium Signaling in Apoptosis-induced Proliferation in Drosophila" by Suthar and Bergmann explores the role of cytosolic calcium influx as a crucial event for DUOX activation during apoptosis-induced proliferation (AiP), using the Drosophila eye disc as a model system. The manuscript is substantially improved and much more complete, with a new way of framing the findings and new data. Some concerns remain, most of which can be addressed without additional experiments.

1. The authors re-used the same control discs in figures 3 and 4 and figures S4 and S7, given that the data are from the same experiment. However, this image re-use gives the impression that there may be only one control disc from each experiment that fits the narrative. Given that the data from these images are quantified, there must be additional control discs that can be used to avoid the re-use.

**Response**: We thank the reviewer for pointing this out and agree that using distinct control images improves the clarity and transparency of the data presentation. Therefore, to avoid any potential misinterpretation, we have now replaced the duplicated control images with representative examples from independent discs within the same experimental set. These updated panels still reflect the quantified data shown in the accompanying graphs and are consistent with the overall phenotype.

2. The initial review requested quantification of the individual phenotypes in the adult heads, which the authors declined to do, maintaining their semiquantitative categories of weak, moderate, and strong. It would be less subjective to quantitate the number of extra bristles per head, percent of heads with ectopic ocelli, percent of heads with reduced eyes, etc.

**Response**: We thank the reviewer for this suggestion and would like to clarify that we did not “decline” to address the original comment. Rather, we clearly defined the overgrowth categories (“weak,” “moderate,” and “severe”) in the legend to Figure 1 and Methods section, and we provided representative examples in Figure 1A with annotated arrows to illustrate the defining features of each category. These categories are applied consistently throughout the manuscript and are based on scoring more than 100 adult flies per genotype across three independent experiments.

While quantifying individual phenotypic features (e.g., extra bristles or ectopic ocelli) could in principle provide additional detail, this would require defining multiple new subcategories and would substantially complicate both the scoring scheme and data presentation. Moreover, since not all flies in the dataset were individually imaged, implementing this alternative analysis would require repeating the experiments. Given that such an analysis would not change the overall conclusions, we believe that the current classification provides a clear, reproducible, and informative representation of the overgrowth phenotypes.

3. Figure 3B and S4A - it is unclear which experiments and controls were conducted at the same time. To be specific, the data for TrpMRNAi, TrpA1RNAi , and Pkd2RNAi in S4A - the original screen - look identical to the data for TrpMRNAi, TrpA1RNAi , and Pdk2RNAi in 3B - the follow-up experiment that tested multiple ways of reducing TrpM and TrpA1 expression. The data for the controls for these two experiments are not identical, but it looks like the data for the three RNAi lines was re-used.

**Response**: We appreciate the reviewer’s attention to detail. In our original submission, we included only the three positive hits from the initial Trp channel RNAi screen, TrpM, TrpA1, and Pkd2, in Figure 3A (now 3B). Because the reviewer requested to see the entire RNAi screen, we expanded the manuscript to include the full RNAi screen in Supplementary Figure S4A of the revised manuscript. As the RNAi data for TrpM, TrpA1, and Pkd2 shown in Figure 3B were originally obtained as part of this screen, we used the same data in both figures for consistency and clarity.

To follow up on these hits, we conducted independent validation experiments using CRISPR/Cas9-generated mutant alleles, which are also shown in Figure 3B. We now represent both sets of data (RNAi and CRISPR/Cas9) in Figure 3B along with their own matched controls. We hope that this clarification and the updated figure fully address the reviewer’s concern.

4. Figure 5 E,F, and G - the phenotype should be quantified across sufficient sample size rather than just showing one disc, especially since the phenotype is so subtle. One method of quantification could be the difference in the number of rows of ommatidia between the dorsal and ventral halves of each eye disc.

**Response**: We appreciate the reviewer’s helpful suggestion. In response, we have quantified the phenotype by measuring the ratio between the dorsal and ventral halves of each eye disc. Since *hid* expression and subsequent regeneration occur specifically in the dorsal domain, the ventral half provides a reliable internal control for assessing regenerative output. These quantifications, now presented in Figure 5H, confirm that the regenerative response is partially impaired when either TrpA1 or RyR activity is reduced. We believe this analysis provides a more objective and robust measure of the subtle phenotype, as the reviewer recommended.

5. The authors should include a more detailed explanation of the methodology used to quantify the calcium flashes. For example, what constitutes a flash? Signal over a specific magnitude or of a specific duration? This question is raised because in Figure 2H, for the ey>p35 genotype, there is a disc that had 9 flashes and one that had 4 flashes, but none of the traces in 2F/S3B appear to have flashes like those in 2G/S3C, even though the quantification claims that two of them did.

**Response**: We thank the reviewer for pointing this out. Upon rechecking our dataset, we discovered that the disc showing 9 flashes had been inadvertently included in the *ey>p35* control group. This was an error, and we have now corrected it by moving the disc into the appropriate group, *ey>hid,p35*. The figure and quantification have been updated accordingly.

Regarding the definition of what constitutes a Ca^2+^ flash, we have defined this in the Methods section as follows (page 19, line 521ff):

*Ca^2+^ flashes were manually quantified from time-lapse GCaMP6s recordings consisting of 600 frames per disc (10-minute duration). For each recording, fluorescence intensity was examined across the entire movie. Ca^2+^ flashes were defined as transient, sharp increases in GCaMP6s fluorescence that was clearly distinguishable from background fluctuations. These events manifest as brief, high-amplitude peaks in intensity traces and short-lived bright spots in the imaging field. Due to variability in spontaneous flash amplitudes across discs,* ***no fixed intensity threshold was applied****. Instead, flashes were identified based on morphological criteria: rapid rise in fluorescence, short duration, and return to baseline. Minor fluctuations or slow drifts in signal were excluded. The* ***total number of visually identifiable flashes*** *was recorded for each disc.*

And in the legend of Figure 2E-G (lines 976-979):

*Ca²⁺ flashes refer to transient, localized increases in intracellular calcium levels, detected as brief bursts of elevated GCaMP6s fluorescence. These flashes are characterized by a rapid onset, short duration, and return to baseline, indicating discrete calcium signaling events within imaginal disc cells.*

We hope this correction and clarification addresses the reviewer’s concern.

6. It is unclear why the mock RNAi is the appropriate control for the Cas9 knockouts or overexpression of Duox deltaEF and other non-RNAi conditions.

**Response**: We appreciate the reviewer’s concern and have clarified the controls throughout the revised manuscript. Specifically, in Figures 1B, 3B, S2B, and S5B, we now consistently include the appropriate genetic control *- ey>hid,p35/+* -for each experimental condition. This genotype represents the standard control for all non-RNAi experiments. Notably, this *ey>hid,p35/+* control produces phenotypes that are virtually indistinguishable from those observed with the previously used mock RNAi control (UAS-luciferase RNAi), further validating its appropriateness.

7. The authors use jargon that lacks precision when describing experimental conditions, such as the terms "undead head capsule overgrowth" line 164, "moderately strong suppressed undead overgrowth" line 259, "undead head" line 904, etc.

**Response**: We thank the reviewer for pointing out areas where greater clarity was needed. In response, we have revised the language in the manuscript to improve precision and avoid ambiguous or informal terms:

**line 164 (now 163)**: “undead head capsule overgrowth” was revised to **“undead overgrowth in** *ey>hid,p35* animals” to specify the genotype and avoid anatomical jargon.

**line 259 (now 258)**: “moderately strong suppressed undead overgrowth” was changed to “moderately suppressed the undead overgrowth phenotype of…”, which more clearly describes the observed phenotype.

**line 904 (now 916)** (title of Figure 1): “undead head” was replaced with “Loss of EF-hand function of DUOX suppresses undead overgrowth and all AiP markers”, providing a clearer and more descriptive summary of the figure’s content.

8. Line 299 describes data not shown - if PLOS Biology has a policy that all data must be shown, these data should be included.

**Response**: These data are now shown in Supplementary Figure S8E,F.

9. For the DuoxEFm mutation, it is unclear if it is heterozygous or homozygous in the experiments. For example, in Line 928 it is written as heterozygous, but is not indicated as heterozygous throughout Figure 1 or S1 labels or line 931, 934, 938, 946, 1131, 1136, 1137, 1141, 1144.

**Response**: As originally noted in the Methods section, the *Duox^EFm^* mutation is homozygous lethal, and all experiments involving this allele were performed using heterozygous animals. To ensure clarity, we have now uniformly indicated the genotype as **Duox^EFm^/+** throughout the text and figure labels, including the relevant lines and figure legends mentioned by the reviewer. We hope this resolves any ambiguity.

10. Figure 3 Legend A and B are reversed. Similar for S2 Legend A and B.

**Response**: Thank you for pointing this out. We have corrected this oversight.

11. Line 1138 - is Wg activity or expression being monitored?

**Response**: It is Wg expression. We corrected this in now line 1156.

12. Line1328 should perhaps read 12h temperature shift.

**Response**: Thank you for catching this typographical error. We have corrected the text to “12h temperature shift” in the revised manuscript (now line 1343). We appreciate the reviewer’s careful reading.

13. Figure 1H-R label each panel with the genotype.

**Response**: While directly labeling each panel with the genotype would make the figure overly busy and potentially detract from the visual information, we agree that clarity is important. To address this, we have restructured the panel designations: panels H–R are now labeled as C′–G′ (middle row) and C’’-G’’ (lower row), corresponding directly to the genotypes shown above panels C–G. This revised labeling scheme ensures that the genotype associated with each image is unambiguously clear without adding visual clutter.

14. Figure 3S-V the notations of statistical significance are too small to see when printed, especially the n.s. in panel V.

**Response**: Thank you for bringing this to our attention. We have increased the font size of all statistical annotations in panels S–V, to ensure they are clearly visible and legible when printed.

15. Figure 4 swapping panels A and B will show the phenotypes before the quantification.

**Response**: Done

16. Figure 5 A - note the time of egg laying and the time of the temperature shift on the schematic.

**Response**: Thank you for this helpful suggestion. We have now clarified the experimental timeline in the legend to Figure 5A and in the Methods section for completeness and reproducibility.
